# Deep Learning Based Analysis of Histopathological Images of Breast Cancer

**DOI:** 10.3389/fgene.2019.00080

**Published:** 2019-02-19

**Authors:** Juanying Xie, Ran Liu, Joseph Luttrell, Chaoyang Zhang

**Affiliations:** ^1^School of Computer Science, Shaanxi Normal University, Xi'an, China; ^2^School of Computing Sciences and Computer Engineering, University of Southern Mississippi, Hattiesburg, MS, United States

**Keywords:** histopathological images, breast cancer, deep convolutional neural networks, autoencoder, transfer learning, classification, clustering

## Abstract

Breast cancer is associated with the highest morbidity rates for cancer diagnoses in the world and has become a major public health issue. Early diagnosis can increase the chance of successful treatment and survival. However, it is a very challenging and time-consuming task that relies on the experience of pathologists. The automatic diagnosis of breast cancer by analyzing histopathological images plays a significant role for patients and their prognosis. However, traditional feature extraction methods can only extract some low-level features of images, and prior knowledge is necessary to select useful features, which can be greatly affected by humans. Deep learning techniques can extract high-level abstract features from images automatically. Therefore, we introduce it to analyze histopathological images of breast cancer via supervised and unsupervised deep convolutional neural networks. First, we adapted Inception_V3 and Inception_ResNet_V2 architectures to the binary and multi-class issues of breast cancer histopathological image classification by utilizing transfer learning techniques. Then, to overcome the influence from the imbalanced histopathological images in subclasses, we balanced the subclasses with Ductal Carcinoma as the baseline by turning images up and down, right and left, and rotating them counterclockwise by 90 and 180 degrees. Our experimental results of the supervised histopathological image classification of breast cancer and the comparison to the results from other studies demonstrate that Inception_V3 and Inception_ResNet_V2 based histopathological image classification of breast cancer is superior to the existing methods. Furthermore, these findings show that Inception_ResNet_V2 network is the best deep learning architecture so far for diagnosing breast cancers by analyzing histopathological images. Therefore, we used Inception_ResNet_V2 to extract features from breast cancer histopathological images to perform unsupervised analysis of the images. We also constructed a new autoencoder network to transform the features extracted by Inception_ResNet_V2 to a low dimensional space to do clustering analysis of the images. The experimental results demonstrate that using our proposed autoencoder network results in better clustering results than those based on features extracted only by Inception_ResNet_V2 network. All of our experimental results demonstrate that Inception_ResNet_V2 network based deep transfer learning provides a new means of performing analysis of histopathological images of breast cancer.

## Introduction

Cancers have become one of the major public health issues. According to statistics by the IARC (International Agency for Research on Cancer) from the WHO (World Health Organization), and GBD (Global Burden of Disease Cancer Collaboration), cancer cases increased by 28% between 2006 and 2016, and there will be 2.7 million new cancer cases emerging in 2030 (Boyle and Levin, 2008; Moraga-Serrano, 2018). Among the various types of cancer, breast cancer is one of the most common and deadly in women (1.7 million incident cases, 535,000 deaths, and 14.9 million disability-adjusted life years) (Moraga-Serrano, 2018). Therefore, the diagnosis of breast cancer has become very important. Although the diagnosis of breast cancers has been performed for more than 40 years using X-ray, MRI (Magnetic Resonance Imaging), and ultrasound etc. (Stenkvist et al., 1978), biopsy techniques are still the main methods relied on to diagnose breast cancer correctly. Common biopsy techniques include fine-needle aspiration, vacuum-assisted biopsy and surgical biopsy. The process involves collecting samples of cells or tissues, fixing them on the microscope slide, and then staining them (Veta et al., 2014). After that, the histopathological images are analyzed and the diagnosis is made by pathologists (Spanhol et al., 2016a).

However, the analysis of the histopathological images is a difficult and time-consuming task that requires the knowledge of professionals. Furthermore, the outcome of the analysis may be affected by the level of experience of the pathologists involved. Therefore, computer-aided (Aswathy and Jagannath, 2017) analysis of histopathological images plays a significant role in the diagnosis of breast cancer and its prognosis. However, the process of developing tools for performing this analysis is impeded by the following challenges. First, histopathological images of breast cancer are fine-grained, high-resolution images that depict rich geometric structures and complex textures. The variability within a class and the consistency between classes can make classification extremely difficult, especially when dealing with multiple classes. The second challenge is the limitations of feature extraction methods for histopathological images of breast cancer. Traditional feature extraction methods, such as scale-invariant feature transform (SIFT) (Lowe, 1999) and gray-level co-occurrence matrix (GLCM) (Haralick et al., 1973), all rely on supervised information. Furthermore, prior knowledge of data is needed to select useful features, which makes the feature extraction efficiency very low and the computational load very heavy. In the end, the final extracted features are only some low-level and unrepresentative features of histopathological images. Consequently, this can lead to the final model producing poor classification results.

Deep learning techniques have the power to automatically extract features, retrieve information from data automatically, and learn advanced abstract representations of data. They can solve the problems of traditional feature extraction and have been successfully applied in computer vision (He et al., 2015; Xie et al., 2018), biomedical science (Gulshan et al., 2016; Esteva et al., 2017) and many other fields.

In view of the powerful feature extraction advantages of deep learning and the challenges in histopathological image analysis of breast cancer, this paper analyzes histopathological images of breast cancer using deep learning techniques. On one hand, we use advanced deep convolutional neural networks, including Inception_V3 (Szegedy et al., 2016) and Inception_ResNet_V2 (Szegedy et al., 2017), combined with transfer learning techniques to classify the histopathological images of breast cancer (Pan and Yang, 2010). On the other hand, by combining deep learning with clustering and utilizing the dimension-reduction functionality of the autoencoder network (Hinton and Salakhutdinov, 2006), we propose a new autoencoder network structure to apply non-linear transformations to features in histopathological images of breast cancer extracted by the Inception_ResNet_V2 network. This effectively maps the extracted features to a lower dimensional space. The newly obtained features are then used as input for the classical clustering algorithm known as K-means (MacQueen, 1967) to perform clustering analysis on histopathological images of breast cancer. Also, we designed a number of comparable experiments to verify the validity of our proposed method of histopathological image analysis of breast cancer images based on deep learning techniques.

## Related Works

Breast cancer diagnosis based on image analysis has been studied for more than 40 years, and there have been several notable research achievements in the area. These studies can be divided into two categories according to their methods: one is based on traditional machine learning methods, and the other is based on deep learning methods. The former category is mainly focused on small datasets of breast cancer images and is based on labor intensive and comparatively low-performing, abstract features. The latter category can deal with big data and can also extract much more abstract features from data automatically.

For example, Zhang et al. (2013) proposed a new cascade random subspace ensemble scheme with rejection options for microscopic biopsy image classification in 2012. This classification system consists of two random subspace classifier ensembles. The first ensemble consists of a set of support vector machines which correspond to the K binary classification problems transformed from the original K-class classification problem (K = 3). The second ensemble consists of a Multi-Layer Perceptron ensemble which focuses on rejected samples from the first ensemble. This system was tested on a database composed of 361 images, of which 119 were normal tissue, 102 were carcinoma *in situ*, and 140 were lobular carcinoma or invasive ductal. The authors randomly split the images into training and testing sets, with 20% of each class' images used for testing and the rest used for training. It obtained a high classification accuracy of 99.25% and a high classification reliability of 97.65% with a small rejection rate of 1.94%. In 2013, Kowal et al. (2013) used four clustering algorithms to perform nuclei segmentation for 500 images from 50 patients with breast cancer. Then, they used three different classification approaches to classify these images into benign and malignant tumors. Among 500 images, there were 25 benign and 25 malignant cases with 10 images per case. They achieved classification accuracy between 96 and 100% using a 50-fold cross-validation technique. In the same year, Filipczuk et al. (2013) presented a breast cancer diagnosis system based on the analysis of cytological images of fine needle biopsies to discriminate between benign or malignant biopsies. Four traditional machine learning methods including KNN (K-nearest neighbor with K = 5), NB (naive Bayes classifier with kernel density estimate), DT (decision tree) and SVM (support vector machine with Gaussian radial basis function kernel and scaling factor σ = 0.9) were used to build the classifiers of the biopsies with 25 features of the nuclei. These classifiers were tested on a set of 737 microscopic images of fine needle biopsies obtained from 67 patients, which contained 25 benign (275 images) and 42 malignant (462 images) cases. The best reported effectiveness is up to 98.51%. In 2014, George et al. (2014) proposed a diagnosis system for breast cancer using nuclear segmentation based on cytological images. Four classification models were used, including MLP (multilayer perceptron using the backpropagation algorithm), PNN (probabilistic neural network), LVQ (learning vector quantization), and SVM. The parameters for each model can be found in Table 5 in George et al. (2014). The classification accuracy using 10-fold cross-validation is 76~94% with only 92 images, including 45 images of benign tumors and 47 images of malignant tumors. In 2016, a performance comparison was conducted by Asri et al. (2016) between four machine learning algorithms, including SVM, DT, NB and KNN, on the Wisconsin Breast Cancer dataset, which contains 699 instances (including 458 benign and 241 malignant cases). Experimental results demonstrated that SVM achieved the highest accuracy of 97.13% with 10-fold cross-validation.

However, the above breast cancer diagnosis studies focused on Whole-Slide Imaging (Zhang et al., 2013, 2014). Since the operation of Whole-Slide Imaging is complex and expensive, many studies based on this technique use small datasets and achieve poor generalization performance. To solve these problems, Spanhol et al. (2016a) published a breast cancer dataset called BreaKHis in 2016. BreaKHis contains 7,909 histopathological images of breast cancer from 82 patients. The authors used 6 different feature descriptors and 4 different traditional machine learning methods, including 1-NN (1 Nearest Neighbor), QDA (Quadratic Discriminant Analysis), RF (Random Forest), and SVM with the Gaussian kernel function, to perform binary diagnosis of benign and malignant tumors. The classification accuracy is between 80 and 85% using 5-fold cross-validation.

Although traditional machine learning methods have made great achievements in analyzing histopathological images of breast cancer and even in dealing with relatively large datasets, their performance is heavily dependent on the choice of data representation (or features) for the task they are trained to perform. Furthermore, they are unable to extract and organize discriminative information from data (Bengio et al., 2013). Deep learning methods typically are neural network based learning machines with much more layers than the usual neural network. They have been widely used in the medical field since they can automatically yield more abstract—and ultimately more useful—representations (Bengio et al., 2013). That is, they can extract the discriminative information or features from data without requiring the manual design of features by a domain expert (Spanhol et al., 2016b).

As a consequence, Spanhol et al. (2016b) classified histopathological images of breast cancer from BreaKHis using a variation of the AlexNet (Krizhevsky et al., 2012) convolutional neural network that improved classification accuracy by 4–6%. Bayramoglu et al. (2016) proposed to classify breast cancer histopathological images independently of their magnifications using CNN (convolutional neural networks). They proposed two different architectures: the single task CNN used to predict malignancy, and the multi-task CNN used to predict both malignancy and image magnification level simultaneously. Evaluations were carried out on the BreaKHis dataset, and the experimental results were competitive with the state-of-the-art results obtained from traditional machine learning methods.

However, the above studies on the BreaKHis dataset only focus on the binary classification problem. Multi-class classification studies on histopathological images of breast cancer can provide more reliable information for diagnosis and prognosis. As a result, Araújo et al. (2017) proposed a CNN based method to classify the hematoxylin and eosin stained breast biopsy images from a dataset composed of 269 images into four classes (normal tissue, benign lesion, *in situ* carcinoma and invasive carcinoma), and into two classes (carcinoma and non-carcinoma), respectively. An SVM classifier with the radial basis kernel function was trained using the features extracted by CNN. The accuracies of the SVM for the four-class and two-class classification problems are 77.8–83.3%, respectively. To realize the development of a system for diagnosing breast cancer using multi-class classification on BreaKHis, Han et al. (2017) proposed a class structure-based deep convolutional network to provide an accurate and reliable solution for breast cancer multi-class classification by using hierarchical feature representation. Using these techniques, they were able to achieve multi-class classification of breast cancer with a maximum accuracy of 95.9%. This study is important for precise treatment of breast cancer. In addition, Nawaz et al. (2018) presented a DenseNet based model for multi-class breast cancer classification to predict the subclass of the tumors. The experimental results on BreaKHis achieved the accuracy of 95.4%. After that, Motlagh et al. (2018) used the pre-trained model of ResNet_V1_152 (He et al., 2016) to perform diagnosis of benign and malignant tumors as well as diagnosis based on multi-class classification of various subtypes of histopathological images of breast cancer in BreaKHis. They were able to achieve an accuracy of 98.7–96.4% for binary classification and multi-class classification, respectively.

Although there are 7,909 histopathological images from 82 patients in BreaKHis, the number of images is far from enough for effectively using deep learning techniques. Therefore, we proposed to combine transfer learning techniques with deep learning to perform breast cancer diagnosis using the relatively small number of histopathological images (7,909) from the BreaKHis dataset.

The Inception_V3 (Szegedy et al., 2016) and Inception_ResNet_V2 (Szegedy et al., 2017) networks were proposed by Szegedy et al. (2016, 2017), respectively. In the 2012 ImageNet Large Scale Visual Recognition Challenge (ILSVRC) competition, the Inception_V3 network achieved 78.0–93.9% accuracy in top-1 and top-5 metrics, respectively, while the Inception_ResNet_V2 achieved 80.4–95.3% accuracy in the same evaluation.

One common method for performing transfer learning (Pan and Yang, 2010) involves obtaining the basic parameters for training a deep learning model by pre-training on large data sets, such as ImageNet, and then using the data set of the new target task to retrain the last fully-connected layer of the model. This process can achieve good results even on small data sets.

Therefore, we adopt two deep convolutional neural networks, specifically Inception_V3 and Inception_Resnet_V2, to study the diagnosis of breast cancer in the BreaKHis dataset via transfer learning techniques. To solve the unbalanced distribution of samples of histopathological images of breast cancer, the BreaKHis dataset was expanded by rotation, inversion, and several other data augmentation techniques. The Inception_ResNet_V2 network was chosen to conduct binary and multi-class classification diagnosis on the expanded set of histopathological breast cancer images for its better performance on the original dataset of BreaKHis compared to that of Inception_V3. The powerful feature extraction capability of the Inception_ResNet_V2 network was used to extract features of the histopathological images of breast cancer for the linear kernel SVM and 1-NN classifiers. The image features extracted by the Inception_ResNet_V2 network are also used as the input of the K-means algorithm to do clustering analysis for the BreaKHis dataset. Furthermore, a new autoencoder deep learning model is constructed to apply a non-linear transformation to the image features extracted by Inception_ResNet_V2 network in order to get the low-dimensional features of the image, and to do clustering analysis for BreaKHis dataset using the K-means algorithm.

## Data and Methods

### Datasets

The dataset named BreaKHis used in this article was published by Spanhol et al. (2016a) in 2016. It is composed of 7,909 histopathological images from 82 clinical breast cancer patients. The database can be accessed through the link http://web.inf.ufpr.br/vri/breast-cancer-database. To save the original organization structure and molecular composition, each image was taken by a pathologist from a patient's breast tissue section using a surgical biopsy. Then, the images were collected via haematoxylin and eosin staining. Finally, the real class label was given to each image by pathologists via their observations of the images from a microscope. All the histopathological images of breast cancer are 3 channel RGB micrographs with a size of 700 × 460. Since objective lenses of different multiples were used in collecting these histopathological images of breast cancer, the entire dataset comprised four different sub-datasets, namely 40, 100, 200, and 400X. All of these sub-datasets are classified into benign and malignant tumors. Therefore, both benign and malignant tumors have four different subsets. Benign tumors include Adenosis (A), Fibroadenoma (F), Phyllodes Tumor (PT), and Tubular Adenoma (TA). Malignant tumors include Ductal Carcinoma (DC), Lobular Carcinoma (LC), Mucinous Carcinoma (MC), and Papillary Carcinoma (PC). Sample descriptions for the BreaKHis dataset are shown in Table 1.

**Table 1:** Image distribution of different subclasses in different magnification factors.

**Magnification**	**Benign**				**Malignant**				**Total**
	**A**	**F**	**PT**	**TA**	**DC**	**LC**	**MC**	**PC**	
40X	114	253	109	149	864	156	205	145	1,995
100X	113	260	121	150	903	170	222	142	2,081
200X	111	264	108	140	896	163	196	135	2,013
400X	106	237	115	130	788	137	169	138	1,820
Total	444	1,014	453	569	3,451	626	792	560	7,909
#Patients	4	10	3	7	38	5	9	6	82

Since the input sizes of Inception_V3 and Inception_ResNet_V2 networks used in this paper are both 299 × 299, each of the histopathological images of breast cancer must be transformed into a 299 × 299 image to match the required input size of the network structure. Some image preprocessing methods in the TensorFlow framework were used in the transforming process, including cutting the border box, adjusting image size, and adjusting saturation, etc. In this way, a 3-channel image conforming to the input size of the model was generated, and the pixel values of each channel were normalized to the interval of [−1, 1]. In order to ensure the universality of the experimental results in the classification task, the datasets of the four magnification factors were randomly partitioned into training and testing subsets according to the proportion of 7:3.

### Classification Analysis

This subsection will discuss our experiments of classifying histopathological images of breast cancer using the deep learning models of Inception_V3 (Szegedy et al., 2016) and Inception_ResNet_V2 (Szegedy et al., 2017) as well as the analyses of our experimental results.

#### Network Structures for Classification

**Figure 1:**
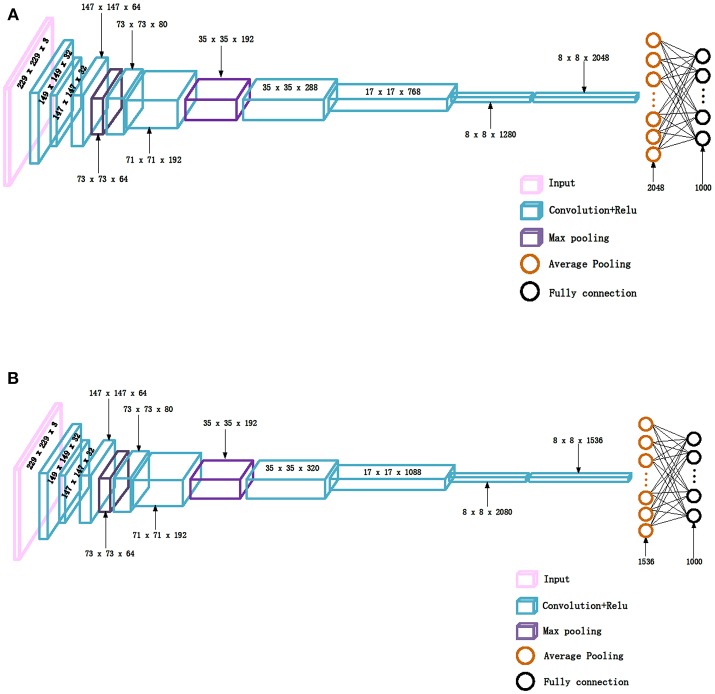
The network structures, **(A)** Inception_V3, **(B)** Inception_ResNet_V2.

The Inception_V3 (Szegedy et al., 2016) and Inception_ResNet_V2 (Szegedy et al., 2017) networks, proposed by Szegedy et al. in 2016 and 2017, respectively, were adopted in our experiments. It was demonstrated in the ILSVRC competition that Inception_ResNet_V2 could defeat the Inception_V3 network when applied to big data. An important difference between the Inception_V3 and Inception_ResNet_V2 networks is that the latter is equipped with residual connections. To test whether the experimental results from Inception_ResNet_V2 are superior to those from Inception_V3 on small datasets or not, these two networks are adopted in this paper to perform classification of the histopathological images of breast cancer. The network structures are shown in Figure 1.

**Figure 2:**
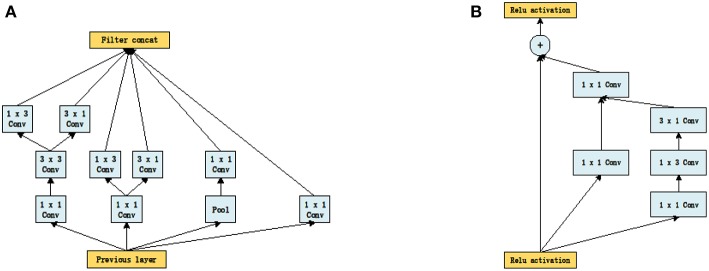
The inception module of size 8 × 8 in two networks, **(A)** Inception_V3, **(B)** Inception_ResNet_V2.

It can be seen from Figure 1 that the structures of the two networks are very similar. The first several layers are characteristic transformation via the traditional convolutional layers and the pooling layers, and the middle part is composed of multiple Inception modules stacked together. The results are finally output through the fully-connected layer using the Softmax function. One of the main differences between the Inception_V3 and Inception_ResNet_V2 networks lies in the differing composition of the two networks' Inception modules. To enhance the network's adaptability to different convolution kernels, each Inception module of the Inception_V3 network is composed of filters with different sizes including 1 × 1, 1 × 3, 3 × 1. For the Inception_ResNet_V2 network, to avoid the deterioration of the network gradient that is often associated with an increase in the number of layers, a residual unit is added to each Inception module. Besides using filters of different sizes in the network, the deterioration caused by increasing layers can also be solved by jumping layers as allowed by the use of residual connections. Figure 2 displays the differences in the construction of the Inception module with a size of 8 × 8 between Inception_V3 and Inception_ResNet_V2. The other details can be found in the original references (Szegedy et al., 2016, 2017).

#### Transfer Learning

Transfer learning (Pan and Yang, 2010) emerges from deep learning. It is well-known that it is typically impossible to train a complex deep network from scratch with only a small dataset. Furthermore, there are not any existing principles to design a network structure for a specific task. What we can do is adopt the model and the parameters obtained by other researchers via time-consuming and computationally intensive training on the very large image dataset of ImageNet and use the knowledge it has gained as pre-training for our specific research task. Then, we can retrain the last defined fully-connected layer of the model using only a relatively small amount of data to achieve good results for our target task.

**Figure 3:**
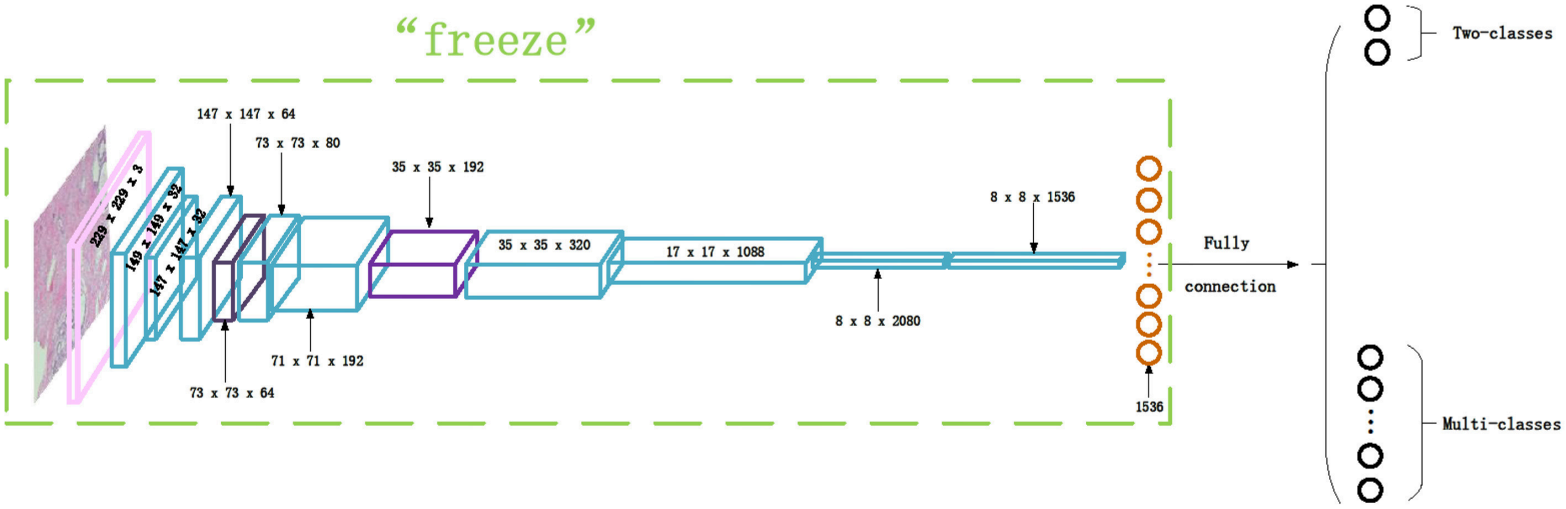
The Inception_ResNet_V2 network structure for transfer learning.

Transfer learning is adopted in this paper to classify the histopathological images of breast cancer using Inception_V3 and Inception_ResNet_V2 networks. We first downloaded the models and parameters of Inception_V3 and Inception_ResNet_V2 networks trained on the ImageNet dataset. The dataset is composed of about 1.2 million training images, 50,000 validation images, and 100,000 testing images. This comprises a total of 1,000 different categories. Then, we froze all of the parameters before the last layer of the networks. We modified the number of neurons of the last fully-connected layer as 2 for binary classification and 8 for multi-class classification. After that, the parameters of the fully-connected layer are trained on the histopathological images of breast cancer. The modified network structure of the Inception_ResNet_V2 network is shown in Figure 3. The modified Inception_V3 network structure is similar, so it is omitted.

Our classification process was developed based on the TensorFlow deep learning framework. The Adam (adaptive moment estimation) (Kingma and Ba, 2014) algorithm was used in the training process to perform optimization by iterating through 70 epochs using the histopathological image dataset of breast cancer. The batch_size is set to 32 in the experiments, and the initial learning rate is 0.0002 (Bergstra and Bengio, 2012). Then, the exponential decay method is adopted to reduce the learning rate and ensure that the model moves through iterations quickly at the initial training stage. This also helps to provide more stability at the later stage and makes it easier to obtain the optimal solution. The decay coefficient is set as 0.7 (Bergstra and Bengio, 2012), and the decay speed is set so that the decay occurs every two epochs. The specific decay process is shown in (1), where *decayed*_*learning*_*rate* is the current learning rate, *learning*_*rate* is the initial learning rate, *decay*_*rate* is the decay coefficient, *global*_*step* is the current iteration step, and *decay*_*steps* is the decay speed.

(1) $$\begin{array}{l} decayed\text{\_}learning\text{\_}rate=learning\text{\_}rate\\ \;\times decay\text{\_}rate^{\left(global\text{\_}step/decay\text{\_}steps\right)}\; \end{array}$$

#### Evaluation Criteria for Classification Results

To evaluate the performance of the classification model more accurately and comprehensively, the classification results are evaluated by some popular benchmark metrics, including sensitivity (Se), specificity (Sp), positive predictive value (PPV), diagnostic odds ratio (DOR), F1 measure (F1), area under the receiver operating characteristic curve (AUC), Kappa criteria (Kappa), Macro-F1, Micro-F1, image level test accuracy (ACC_IL), and patient level test accuracy (ACC_PL). The latter two criteria were proposed in (5). The Macro-F1 and Micro-F1 are two variations of F1 for multi-class classification problems. Macro-F1 is the average of F1 for each class. Micro-F1 is defined as F1 but depending on the precision and recall defined by the sum of TP (true positive), FP (false positive), and FN (false negative) for all classes. The definitions of the criteria are shown in Equations (2–9).

(2) $$\begin{array}{lll} Se & = & \frac{TP}{TP+FN} \end{array}$$

(3) $$\begin{array}{lll} Sp & = & \frac{TN}{TN+FP} \end{array}$$

(4) $$\begin{array}{lll} PPV & = & \frac{TP}{TP+FP} \end{array}$$

(5) $$\begin{array}{lll} DOR & = & \frac{TP\times TN}{FP\times FN} \end{array}$$

(6) $$\begin{array}{lll} ACC\text{\_}IL & = & \frac{N_{rec}}{N_{all}} \end{array}$$

(7) $$\begin{array}{lll} ACC\text{\_}PL & = & \frac{\sum Patient\;Score}{Total\;Number\;of\;Patients},\;Patient\;Score=\frac{N_{rec}}{N_{P}} \end{array}$$

(8) $$\begin{array}{lll} F1 & = & \frac{2\times precision\times recall}{precision+recall},\;recall=\frac{TP}{TP+FN} \end{array}$$

(9) $$\begin{array}{lll} Kappa & = & \frac{p_{0}-p_{e}}{1-p_{e}},p_{0}=\frac{N_{rec}}{N_{all}},P_{e}=\frac{\sum N_{true\text{\_}i}\times N_{pre\text{\_}i}}{N_{all}\times N_{all}} \end{array}$$

The value of TP in the equations above is the number of images correctly recognized as malignant tumor in the testing subset. FP is the number of images that were incorrectly recognized as malignant tumor in the testing subset. FN is the number of images incorrectly recognized as benign tumor in the testing subset. TN is the number of images correctly recognized as benign tumor in the testing subset. Therefore, Se in (2) defines the ratio of the recognized malignant tumor images to all malignant tumor images in the testing subset. Sp in (3) expresses the ratio of the recognized benign tumor images to all benign tumor images. That is, Se and Sp are the accuracy of the positive and negative class, respectively. PPV in (4) is the ratio of correctly recognized malignant tumor images to all recognized malignant tumor images in the testing subset. In fact, it is the precision in (8). DOR expresses the ratio of the product of TP and TN to the product of FP and FN. It is clear that DOR will become infinity when the related classifier is perfect. It is reported that a diagnosis system is reliable if Se> = 80%, Sp> = 95%, PPV> = 95%, and DOR> = 100 (Ellis, 2010; Colquhoun, 2014). Equation (6) defines image level test accuracy (ACC_IL) by the ratio of *N*_*rec*_ (the number of the histopathological images of breast cancer correctly identified in the testing subset), to *N*_*all*_ (the total number the histopathological images of breast cancers in the testing subset). Equation (7) defines patient level test accuracy (ACC_PL), that is, the ratio of the sum of patient score to the total number of patients in the testing subset. Here, the patient score is the ratio of *N*_*rec*_ to *N*_*P*_, that is, the ratio of correctly identified images of patient P to all the images of patient P in the testing subset. Equation (8) describes a popular metric known as the harmonic mean of precision and recall. Here, precision is the same as PPV defined as the ratio of correctly recognized malignant tumor images to all recognized malignant tumor images in the testing subset, and recall is the ratio of correctly recognized malignant tumor images to the true number of malignant tumor images in the testing subset. AUC is the area under the ROC curve, which is another widely used metric for evaluating binary classification models. The range of AUC is [0, 1] (Bradley, 1997), with higher values representing better model performance. We calculate AUC in our experiments by calling the roc_auc_score function from the Scikit-learn library that is available as a Python package (sklearn). Equation (9) is the Kappa coefficient, where *P*_0_ is the image level test accuracy defined in (6), and *P*_*e*_ is the ratio of the sum of the product of the number of real images in each category and the predicted number of images in that category to the square of the total samples. The calculation of the Kappa coefficient is based on the confusion matrix. Kappa is used for consistency checking, and its value is in the range of [−1, 1]. It can be divided into six groups representing the following consistency levels: −1~0.0 (poor), 0.0~0.20 (slight), 0.21~0.40 (fair), 0.41~0.60 (moderate), 0.61~0.80 (substantial), and 0.81~1 (almost perfect) (Landis and Koch, 1977).

### Clustering Analysis

The classification analysis of histopathological images of breast cancer based on deep convolutional neural networks is introduced in the previous section. However, this type of classification is supervised learning and requires experienced pathologists to examine the histopathological images of breast cancer and assign labels to them that identify the data as coming from patients or normal people. This is very difficult, time-consuming, and expensive work, especially with the increasing number of samples in the dataset. On the contrary, unsupervised learning, specifically clustering, does not need any labels for samples. It only uses the similarities between samples to group them into different clusters, such that the samples in the same cluster are similar to each other and dissimilar to those from other clusters. Therefore, we adopt clustering techniques to study the histopathological images of breast cancer.

#### Network Structures for Clustering

The Inception_ResNet_V2 network is adopted to extract features for performing clustering analysis of the histopathological images of breast cancer because of its excellent performance when classifying these images using its advantage of extracting features automatically. Each histopathological image of breast cancer can be well-expressed by the extracted features of the 1,536-dimension vector produced by the Inception_ResNet_V2 network before its final classification layer. The extracted feature vectors are used as input to a clustering algorithm in order to perform clustering analysis on the histopathological images of breast cancer.

**Figure 4:**
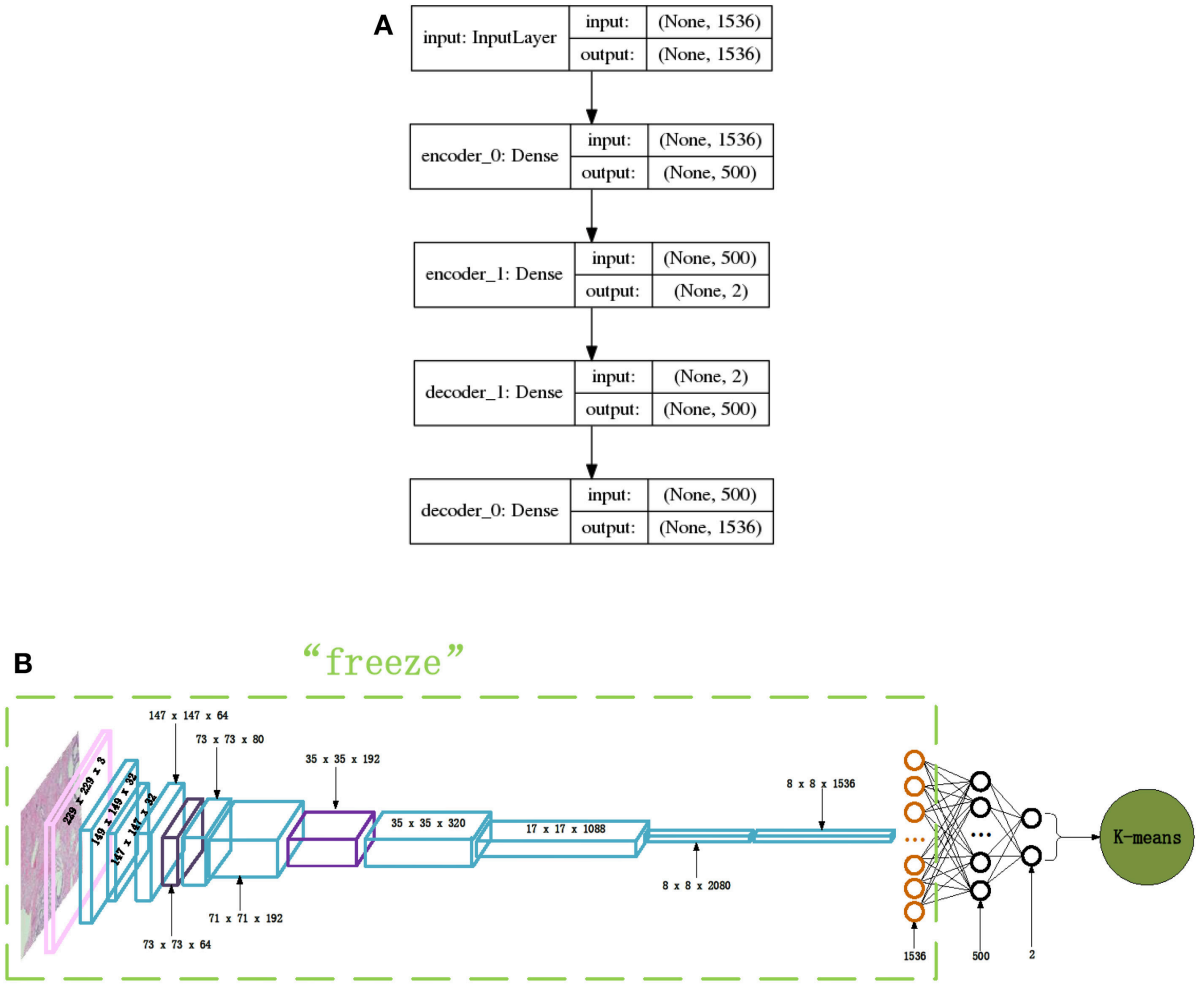
The network structures of our proposed autoencoder and its combination with Inception_ResNet_V2, **(A)** Autoencoder network, **(B)** Inception_ResNet_V2 and autoencoder network.

The very simple and fast, typical clustering algorithm K-means is adopted in this paper to perform this clustering analysis. To determine the proper value of K for the K-means algorithm, the internal criterion metric SSE (Silhouette Score) (Rousseeuw, 1987) is adopted to search for the optimal K. The features extracted by the Inception_ResNet_V2 network for each breast cancer histopathological image are thought of as a representation of the images, and the K-means clustering algorithm is adopted to cluster the breast cancer histopathological images into clusters. Also, in order to get better clustering results and to visualize the clustering results, we constructed a new autoencoder network to map the 1,536-dimension vector to a 2-dimension vector via a non-linear transformation. In this way, the breast cancer histopathological images can be represented in a very low dimensional space. Figure 4A displays the autoencoder network we constructed in our experiments. There are 2 encode layers with neuron sizes of 500 and 2, respectively, and there are 2 corresponding decode layers to reconstruct the original input. Using this autoencoder, the 1,536-dimension feature vector extracted by the Inception_ResNet_V2 network for a breast cancer histopathological image will be transformed to 2-dimenision feature vector via training the layers depicted in Figure 4A. Then, the 2-dimension feature vector is used as input for K-means which performs the clustering analysis for histopathological images of breast cancer. The entire network is shown in Figure 4B.

#### Evaluation Criteria of Clustering Results

The evaluation criteria of clustering results comprise internal and external metrics. The internal metrics are independent of the external information, so they are always used to find the true number of clusters in a dataset. The external metrics depend on the true pattern of the dataset. Some of the most common external metrics are clustering accuracy (ACC), adjusted rand index (ARI) (Hubert and Arabie, 1985), and adjusted mutual information (AMI) (Vinh et al., 2010).

The internal metric SSE (Silhouette Score) (Rousseeuw, 1987) is used in our experiments. It is first used to find the most proper number of clusters of the histopathological images of breast cancer. Then, after the clustering results have been obtained by K-means, it is used to evaluate the clustering results together with the aforementioned external metrics. Equation (10) gives the Silhouette value of sample ***i***.

(10) $$\begin{array}{l} s\left(\text{i}\right)=\frac{b\left(\text{i}\right)-a\left(\text{i}\right)}{\max\left\{a\left(\text{i}\right),b\left(\text{i}\right)\right\}} \end{array}$$

Here, *b*(**i**) is the smallest average distance of sample **i** to all samples in any other cluster to which sample **i** does not belong. *a*(**i**) is the mean distance from sample **i** to all other samples within the same cluster, and *s*(**i**) is the Silhouette value of sample **i**. The average *s*(**i**) of all samples in a cluster is a measure of how tightly grouped all the samples in the cluster are. Therefore, the average *s*(**i**) over all samples in an entire dataset is a measure of how appropriately the samples have been clustered; that is what is called the SSE metric.

The external metrics used in this paper are ACC, ARI (Hubert and Arabie, 1985) and AMI (Vinh et al., 2010). It was reported that ARI is one of the best external metrics (Hubert and Arabie, 1985). ARI is defined in (11) and uses the following variables: *a* (the number of pairs of samples in the same cluster before and after clustering), *b* (the pairs of samples in the same cluster while partitioned into different clusters by the clustering algorithm), *c* (the pairs of samples that are from different clusters but are grouped into the same cluster incorrectly by the clustering algorithm), and *d* (the number of pairs of samples from different clusters that are still in different clusters after clustering). The AMI is defined in (12), where *U* is the original partition and *V* is the clustering of a clustering algorithm. Here, *MI*(*U, V*) denotes the mutual information between two partitions *U* and *V*, and *E*{*MI*(*U, V*)} represents the expected mutual information between the original partition *U* and the clustering *V*. *H*(*U*), *H*(*V*) are the entropy of the original partition *U* and the clustering *V*, respectively. AMI is a variation of mutual information and can be used to compare the clustering *V* of a clustering algorithm and the true pattern *U* of the dataset. It corrects the effect of agreement solely due to chance between the clustering and the original pattern. This is similar to the way that the adjusted Rand index corrects the Rand index.

(11) $$\begin{array}{l} ARI=\frac{2\left(ad-bc\right)}{\left(a+b\right)\left(b+d\right)+\left(a+c\right)\left(c+d\right)} \end{array}$$

(12) $$\begin{array}{l} AMI\left(U,V\right)=\frac{MI\left(U,V\right)-E\left\{MI\left(U,V\right)\right\}}{\max\left\{H\left(U\right),H\left(V\right)\right\}-E\left\{MI\left(U,V\right)\right\}} \end{array}$$

We calculate the criteria listed above in our experiments by calling functions embedded in the sklearn library (available as a Python package), such as silhouette_score (SSE), linear_assignment (ACC), adjusted_rand_score (ARI), and adjusted_mutual _info_score (AMI).

## Experimental Results and Analysis

The section will present our classification and clustering experimental results on the 7,909 histopathological images of breast cancer from the BreaKHis dataset and provide some analyses and discussions of the results.

### Classification Results

This subsection will present and discuss all of the classification results of histopathological images of breast cancer from BreaKHis dataset provided by Spanhol (Spanhol et al., 2016a). The experimental results include those conducted on the raw dataset and on the augmented dataset. In addition to this, we provide a comparison between our results and the results produced by other researchers.

#### Experiments on the Raw Dataset

We used the Inception_V3 and Inception_ResNet_V2 networks to perform binary classification of histopathological images of breast cancer into benign and malignant tumors via transfer learning. Table 2's upper part gives the experimental results using Inception_V3 and Inception_ResNet_V2 networks to perform binary classification on the histopathological images of breast cancer in terms of Se, Sp, PPV, DOR, ACC_IL, ACC_PL, F1, AUC and Kappa. In the table, INV3 is the abbreviation for the Inception_V3 network, and IRV2 is the abbreviation for the Inception_ResNet_V2 network.

**Table 2:** Results of binary and multi-class classification using Inception_V3 (INV3) and Inception_ResNet_V2 (IRV2)/%.

**Classification**	**Network**	**Criteria**	**Magnification factors**			
			**40X**	**100X**	**200X**	**400X**
Binary	INV3	Se	98.00	98.48	**99.01**	96.41
		Sp	**94.31**	93.46	91.40	90.99
		PPV	**97.41**	96.67	95.88	95.89
		DOR	81,233	92,303	**106,700**	27,105
		ACC_IL	**96.84**	96.76	96.49	94.71
		ACC_PL	**97.74**	94.19	87.21	96.67
		F1	**97.70**	97.56	97.42	96.15
		AUC	**99.47**	99.03	99.29	97.91
		Kappa	92.64	**92.74**	91.95	87.68
	**IRV2**	Se	98.48	98.90	99.13	98.06
		Sp	96.63	92.95	92.80	92.10
		PPV	98.46	96.45	96.39	96.51
		DOR	185,774	118,782	147,138	58,835
		ACC_IL	97.90	96.88	96.98	96.98
		ACC_PL	**98.03**	97.07	82.74	88.12
		F1	98.47	97.66	97.74	97.28
		AUC	99.57	98.84	99.61	98.81
		Kappa	95.12	92.96	93.18	91.05
Multi-class	INV3	ACC_IL	**90.28**	85.35	83.99	82.08
		ACC_PL	90.44	89.05	80.63	81.08
		Macro-F1	**88.55**	82.59	79.64	77.98
		Micro-F1	**90.28**	85.35	83.99	82.08
		Kappa	**87.37**	80.26	77.91	76.39
	**IRV2**	ACC_IL	92.07	88.06	87.62	84.50
		ACC_PL	**89.11**	88.45	86.07	71.42
		Macro-F1	90.89	85.67	84.08	80.13
		Micro-F1	92.07	88.06	87.62	84.50
		Kappa	89.74	84.03	82.84	79.70

*† For each magnification factor, the underline shows the best result of each evaluation index between the two network structures of INV3 and IRV2. Bold font shows the best result of each evaluation index with respect to the different magnification factors*.

According to the description of the histopathological image dataset of breast cancer, the benign and malignant tumors can be classified into four different subclasses, respectively. So, there are 8 subclasses in total, including 4 benign tumors (A, F, PT, and TA) and 4 malignant tumors (DC, LC, MC, and PC). The available studies for the histopathological images of breast cancer only focus on binary classification of the images. However, multi-class classification is more significant than binary classification for providing accurate treatment and prognosis for breast cancer patients. Therefore, we did a multi-class classification diagnosis study on the histopathological images of breast cancer by using Inception_V3 and Inception_ResNet_V2 with transfer learning techniques. The experimental results of our multi-class classification of histopathological images of breast cancer are shown in the bottom half in Table 2 in terms of ACC_IL, ACC_PL, Macro-F1, Micro-F1 and Kappa.

The experimental results in Table 2 show that the Inception_ResNet_V2 network can get better results in all evaluation metrics compared to the Inception_V3 network, regardless of binary or multi-class classification (which is indicated by the red underline). One reason for this is that residual connections are added to the Inception_ResNet_V2 network, which avoids the vanishing gradient problem typically caused by increasing the number of layers in a network. This also improves the network performance and allows it to extract more informative features from images than Incepiton_V3 can.

Furthermore, the experimental results show that all metrics on the 40X dataset are better than those on the other datasets with any other magnification factors, which is shown in black font. These results are in agreement with those reported in (5). The reason for this should be the 40X dataset containing more significant characteristics of breast cancer.

The experimental results in Table 2 show that Se>95%, Sp>90%, PPV>95%, and DOR>100 on each dataset regardless of magnification factor and network structure (Inception_V3 or Inception_ResNet_V2). The results from the Inception_ResNet_V2 network show that Se>98%, Sp>92%, PPV>96%, and DOR>100, especially on the 40X dataset where Se >98%, Sp>96%, PPV>98%, and DOR>100. Considering research which suggests that a diagnosis system is reliable when Se> = 80%, Sp> = 95%, PPV> = 95%, and DOR> = 100 (Ellis, 2010; Colquhoun, 2014), we can say that our breast cancer diagnosis system based on the 40X dataset and the Inception_ResNet_V2 network is very reliable. The diagnosis system based on the Incepiton_V3 network is also comparatively reliable.

In addition, the values of AUC and Kappa in Table 2 tell us that our models are perfect and have obtained almost perfect agreement for binary classification of histopathological images of breast cancer. The values of Kappa in Table 2 reveal that our models for multi-class classification are also perfect. The models based on the Inception_ResNet_V2 network can get perfect agreement for multi-class classification of breast cancer histopathological images, except when applied to the 400X dataset (which still achieves substantial agreement).

Besides the above analysis, we further verify the power of our approaches for analyzing the breast cancer histopathological images using the *p*-value of AUC and Kappa. The *p*-value is a probability that measures the statistical significance of evidence against the null hypothesis. A lower *p*-value provides stronger evidence to reject the null hypothesis. Therefore, to determine whether the predictions are due to chance, we calculate the *p*-values for AUC and Kappa and compare the *p*-value to the significance level α. It is usually set as α = 0.05. We consider both binary and multi-class classification of breast cancer histopathological images with Inception_ResNet_V2 when calculating the *p*-value for AUC and Kappa. The null hypothesis is “the prediction is a random guess.” The *p*-values for AUC and Kappa are calculated in Equations (13–16) and the pnorm function in R. It should be noted that for multi-class classification, there is only the *p*-value of Kappa to be calculated.

(13) $$\begin{array}{l} SE_{AUC}=\sqrt{\frac{0.25+\left(na+nn-2\right)}{na\times nn\times 12}} \end{array}$$

(14) $$\begin{array}{l} Z_{AUC}=\frac{A-0.5}{SE_{AUC}} \end{array}$$

(15) $$\begin{array}{l} SE_{Kappa}=\frac{\sqrt{p_{0}\times \left(1-p_{0}\right)}}{\sqrt{N}\times \left(1-p_{e}\right)} \end{array}$$

(16) $$\begin{array}{l} Z_{Kappa}=\frac{Kappa}{SE_{Kappa}} \end{array}$$

Here, *na* and *nn* in (13) are, respectively, the number of abnormal (malignant tumor) and normal (benign tumor) samples (breast cancer histopathological images) in the testing subset. *A* in (14) is the value of AUC. *p*_0_ and *p*_*e*_ in (15) are the same as those in (9), and N in (15) is the total number of samples. We convert the z value for AUC in (14) and for Kappa in (16) to the corresponding *p*-value by using the pnorm function in R.

Except for in binary classification, the *p*-values for AUC are *p* = 6.88e-85 (40X), *p* = 2.24e-89 (100X), *p* = 3.73e-89 (200X), and *p* = 9.20e-75 (400X). *P*-values for Kappa are all 0.0, regardless of binary or multi-class classification. All the *p*-values for AUC and Kappa are much <0.05. This means that we can reject the null hypothesis (that the predictive result is a random guess) and accept that our prediction is statistically significant and not random. This holds true for both our binary and multi-class image classification results.

#### Experiments on the Augmented Dataset

Comparing the results in Table 2 for binary and multi-class classification, we can see that the performance of multi-class classification is worse than that of the binary classification. So, we output the confusion matrix of multi-class classification for further analysis. The confusion matrix can be found in the Supplementary Material. From observing this confusion matrix, we can see that many benign tumors are incorrectly classified as malignant tumors. This causes a high false positive rate. For example, some samples from F are erroneously recognized as being from DC. Also, the different subclasses in the same class are often misclassified, such as samples from LC being recognized as samples from DC. One reason leading to the poor classification results for multi-class classification is the imbalance in sample distribution. This makes the extracted features unable to thoroughly represent the subclasses with fewer samples. As a result, the samples from the subclass with fewer samples are erroneously classified into the categories with more samples.

To avoid the high false positive rate in multi-class classification, we expanded the original samples of the dataset to suppress the influence that sample imbalance has on the experimental results. For each magnification factor dataset, we chose the DC subclass as the baseline, and amplified each of the remaining subclasses by turning images up and down, left and right, and using counterclockwise rotation of 90°and 180°. After doing this, the sample number of each subclass was approximately the same. The extended datasets are shown in Table 3.

**Table 3:** The augmented image distribution of different subclasses in different magnification factors.

**Magnification**	**Benign**				**Malignant**				**Total**
	**A**	**F**	**PT**	**TA**	**DC**	**LC**	**MC**	**PC**	
40X	798	759	763	894	864	936	1,025	870	6,909
100X	791	780	847	900	903	1,020	1,110	852	7,203
200X	777	792	756	840	896	978	980	810	6,829
400X	742	711	805	780	788	822	845	828	6,321
Total	3,108	3,042	3,171	3,414	3,451	3,756	3,960	3,360	27,262
#Patients	4	10	3	7	38	5	9	6	82

**Figure 5:**
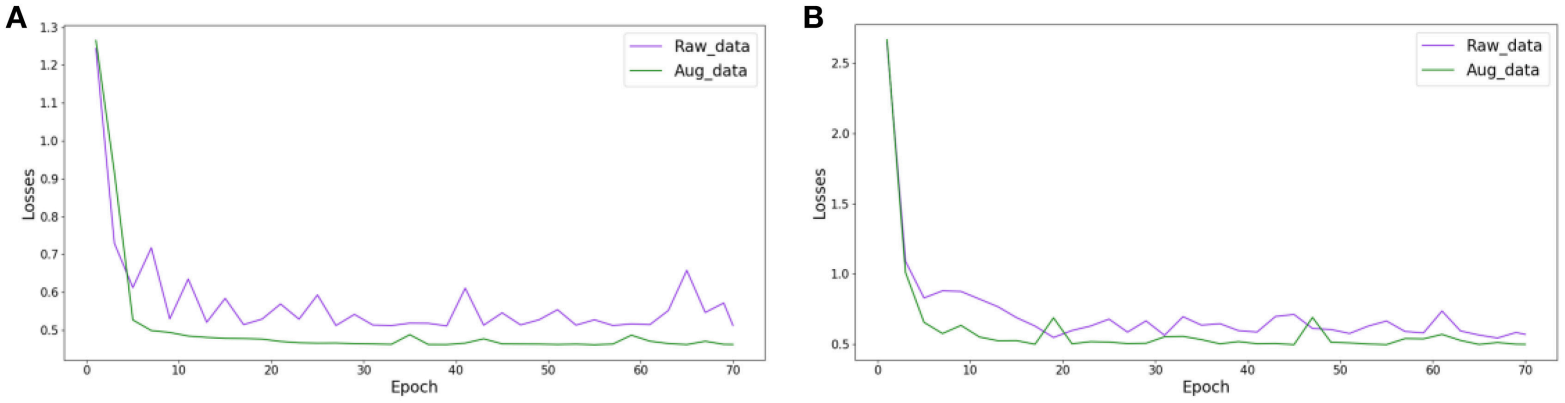
The change in the loss function during the training of Inception_ResNet_V2 on raw and augmented data with 40 factor magnification, **(A)** binary classification, **(B)** multi-class classification.

We randomly partitioned the extended datasets into training and testing subsets in a 7:3 ratio as we did with the original datasets. Then, we used transfer learning to retrain the Inception_ResNet_V2 network to perform effective diagnosis of breast cancer based on histopathological images of breast cancer. Here, we only retrained the Inception_ResNet_V2 network because it performed better than the Incepiton_V3 network on the raw datasets. To compare the differences in the loss function on the original datasets and on the expansion datasets during the training process, we plotted the value of the loss function changing with the number of epochs in the raw and extended datasets. Here, we only compared the loss function from the Inception_ResNet_V2 network on the 40X dataset in order to observe the changing trend of the loss function. The trends of the other magnification factor datasets are similar. Figure 5 compared the loss function of the Inception_ResNet_V2 network on the raw and extended datasets, respectively, for binary and multi-class classification of histopathological images of breast cancer. Table 4 shows the experimental results on the original and expanded datasets for binary and multi-class classification, respectively. The deep learning parameters for both binary and multi-class classification remain the same.

**Table 4:** Results of binary and multi-class classification on raw and augmented data using Inception_ResNet_V2/%.

**Classification**	**Datasets**	**Criteria**	**Magnification factors**			
			**40X**	**100X**	**200X**	**400X**
Binary	Raw_data	Se	98.48	98.90	**99.13**	98.06
		Sp	**96.63**	92.95	92.80	92.10
		PPV	**98.46**	96.45	96.39	96.51
		DOR	**185,774**	118,782	147,138	58,835
		ACC_IL	**97.90**	96.88	96.98	96.98
		ACC_PL	**98.03**	97.07	82.74	88.12
		F1	**98.47**	97.66	97.74	97.28
		AUC	99.57	98.84	**99.61**	98.81
		Kappa	**95.12**	92.96	93.18	91.05
	**Aug_data**	Se	99.95	99.45	99.65	98.88
		Sp	99.61	99.26	99.18	99.34
		PPV	99.66	99.39	99.31	99.42
		DOR	56122,884	2440,736	3427,114	1342,245
		ACC_IL	99.79	99.37	99.43	99.10
		ACC_PL	99.93	99.96	**100.0**	99.90
		F1	99.81	99.42	99.48	99.15
		AUC	100.0	99.99	99.95	99.97
		Kappa	99.59	98.72	98.86	98.19
Multi-class	Raw_data	ACC_IL	**92.07**	88.06	87.62	84.50
		ACC_PL	**89.11**	88.45	86.07	71.42
		Macro-F1	**90.89**	85.67	84.08	80.13
		Micro-F1	**92.07**	88.06	87.62	84.50
		Kappa	**89.74**	84.03	82.84	79.70
	**Aug_data**	ACC_IL	97.63	97.00	96.89	97.49
		ACC_PL	98.42	98.07	97.85	97.40
		Macro-F1	97.68	97.06	97.02	97.48
		Micro-F1	97.63	97.00	96.89	97.49
		Kappa	97.28	96.55	96.44	97.13

† The underline shows the best result of each metric between the two network structures being compared (INV3 and IRV2). The bold font shows the best result of each metric for each magnification level.

The results in Figure 5 show that the value of the loss function decreases much faster and more smoothly converges to a much smaller value on the extended datasets than on the raw datasets. This is true for both experiments on binary and multi-class classification of histopathological images of breast cancer.

The experimental results in Table 4 show that the experiments on extended datasets have produced much better results than those performed on the raw datasets. This is reflected by the data marked with red underlines, especially the results of multi-class classification on the expanded datasets. These results are a significant improvement compared to those from the original datasets. In addition, the experimental results in Table 4 tell us that the evaluation metrics of experimental results on 40X datasets are much better than those on any other datasets with different magnification factors, which can also be seen from the values with black fonts in Table 4. The results further demonstrate that the 40X dataset should contain more significant characteristics of breast cancer.

The experimental results in Table 4 for binary classification show that Se>98%, Sp>92%, PPV>96%, and DOR>100 on each dataset regardless of magnification factor or the effects of augmentation (raw or augmented). This is especially true for the results on the augmented datasets where Se>98%, Sp>99%, PPV>99%, and DOR>100. This tells us that the breast cancer diagnosis system based on the augmented dataset and the Inception_ResNet_V2 network is very reliable. Compared to the results in Table 2, we can say that augmenting raw imbalanced breast cancer histopathological image datasets can greatly improve the reliability of the diagnosis system.

In addition, the values of AUC in Table 4 show that our models are excellent. One even achieved the maximum value of AUC (1.0) on the augmented 40X dataset. The values of Kappa in Table 4 show that our models have obtained perfect agreement for binary classification of histopathological images of breast cancer. The values of Kappa in Table 4 show that our models are perfect when applied to augmented datasets for multi-class classification.

Furthermore, we calculated the *p*-values for AUC and Kappa on all augmented datasets for binary and multi-class classification. The *p*-values for AUC and Kappa are both 0.0, which is much <0.05. This fact tells us that we can reject the null hypothesis (that the prediction result is a random guess), and accept the fact that our prediction is statistically significant and not random.

#### Experimental Comparisons

This subsection will compare the experimental results of classifying histopathological images of breast cancer using the Inception_V3 and Inception_ResNet_V2 networks in addition to a selection of methods from the available studies carried by other research teams. The experimental results will be compared in terms of ACC_IL and ACC_PL, because the available studies only used these two evaluation criteria. The binary and the multi-class classification experimental results are displayed in Table 5. Here, INV3_Raw denotes the results obtained by using Inception_V3 on original dataset. IRV2_Raw and IRV2_Aug represent the results produced by Inception_ResNet_V2 on the original and extended datasets, respectively. The bold fonts denote the best results.

**Table 5:** Binary and multi-class classification comparison between our experimental results and the ones available from other studies /%.

**Classification**	**Criteria**	**Methods**	**Magnification factors**			
			**40X**	**100X**	**200X**	**400X**
Binary	ACC_IL	AlexNet_Raw(25)	85.6 ± 4.8	83.5± 3.9	83.1± 1.9	80.8± 3.0
		CSDCNN_Raw(29)	95.8± 3.1	96.9± 1.9	96.7± 2.0	94.9± 2.8
		INV3_Raw	96.84	96.76	96.49	94.71
		IRV2_Raw	97.90	96.88	96.98	96.21
		**IRV2_Aug**	**99.79**	**99.37**	**99.43**	**99.10**
	ACC_PL	PFTAS+QDA_Raw(5)	83.8± 4.1	82.1± 4.9	84.2± 4.1	82.0± 5.9
		PFTAS+SVM_Raw(5)	81.6± 3.0	79.9± 5.4	85.1± 3.1	82.3± 3.8
		AlexNet_Raw(25)	90.0± 6.7	88.4± 4.8	84.6± 4.2	86.1± 6.2
		CSDCNN_Raw(29)	97.1± 1.5	95.7± 2.8	96.5± 2.1	95.7± 2.2
		INV3_Raw	97.74	94.19	87.23	96.67
		IRV2_Raw	98.03	97.07	82.74	88.12
		**IRV2_Aug**	**99.93**	**99.96**	**100.0**	**99.90**
Multi-class	ACC_IL	LeNet_Raw(29)	40.1± 7.1	37.5± 6.7	40.1± 3.4	38.2± 5.9
		LeNet_Aug(29)	46.4± 4.5	47.3± 4.9	46.5± 5.6	45.2± 9.1
		AlexNet_Raw(29)	70.1± 7.4	68.1± 7.6	67.6± 4.8	67.3± 3.4
		AlexNet_Aug(29)	86.4± 3.1	75.8± 5.4	72.6± 4.8	84.6± 3.6
		CSDCNN_Raw(29)	89.4± 5.4	90.8± 2.5	88.6± 4.7	87.6± 4.1
		CSDCNN_Aug(29)	92.8± 2.1	93.9± 1.9	93.7± 2.2	92.9± 1.8
		INV3_Raw	90.28	85.35	83.99	82.08
		IRV2_Raw	92.07	88.06	87.62	84.50
		**IRV2_Aug**	**97.63**	**97.00**	**96.89**	**97.49**
	ACC_PL	LeNet_Raw(29)	38.1± 9.3	37.5± 3.4	38.5± 4.3	37.2± 3.6
		LeNet_Aug(29)	48.2± 4.5	47.6± 7.5	45.5± 3.2	45.2± 8.2
		AlexNet_Raw(29)	70.4± 6.2	68.7± 5.3	66.4± 4.3	67.2± 5.6
		AlexNet_Aug(29)	74.6± 7.1	73.8± 4.5	76.4± 7.4	79.2± 7.6
		CSDCNN_Raw(29)	88.3± 3.4	89.8± 4.7	87.6± 6.4	87.0± 5.2
		CSDCNN_Aug(29)	94.1± 2.1	93.2± 1.4	94.7± 3.6	93.5± 2.7
		INV3_Raw	90.44	89.05	80.63	81.08
		IRV2_Raw	89.11	88.45	86.07	71.42
		**IRV2_Aug**	**98.42**	**98.07**	**97.85**	**97.40**

*† Bold fonts represent the best results among compared approaches with the same classifier*.

The experimental results in Table 5 tell us that both the evaluation criteria of ACC_IL and ACC_PL applied to the results obtained from the Inception_ResNet_V2 network have the best value among all of the available studies we found in the literature concerning the classification of histopathological images of breast cancer on the expanded datasets for both binary and multi-class classification. The results on the raw datasets produced by the Inception_ResNet_V2 network are better than those produced by other networks. Therefore, the deep learning network of Inception_ResNet_V2 with residual connections is very suitable for classifying the histopathological images of breast cancer. Also, using the expanded histopathological image datasets of breast cancer can obtain better classification and diagnosis results.

To judge whether or not our approaches are statistically significant, we adopted the Friedman's test (Borg et al., 2013) to discover the significant difference between the compared algorithms. If a significant difference has been detected by Friedman's test, then the multiple comparison test is used as a *post hoc* test to detect the significant difference between pairs of the compared algorithms. Friedman's test is considered preferable for comparing algorithms over several datasets without any normal distribution assumption (Borg et al., 2013). We conducted Friedman's test at α = 0.05 using the results of algorithms on all datasets in terms of ACC_IL and ACC_PL for binary and multi-class classification shown in Table 5. The Friedman's test results are shown in Table 6. Here, χ^2^ is chi-square, *df* is the degree of freedom, and *p* is *p*-value.

**Table 6:** Results of Friedman's test between our approaches and the compared algorithms atα = 0.05.

	**Binary classification**			**Multi-class classification**		
	****χ**^**2**^**	**df**	**p**	****χ**^**2**^**	**df**	**p**
ACC_IL	14.6	4	0.0056	30.6667	8	0.0002
ACC_PL	18.1071	6	0.0060	30.8667	8	0.00015

The Friedman's test results in Table 6 tell us that there is a strong significant difference between our approaches and the compared algorithms because any *p* in Table 6 supports *p* ≺ 0.05. Therefore, we conduct a multiple comparison test between each pair of algorithms at the confidence level of 0.95 and show these statistical test results in Table 7. The mean rank difference between algorithms is shown in the upper triangle of the table. The statistical significance between pairs of algorithms is displayed in the lower triangle using “^*^.”

Table 7Paired rank comparison of algorithms in ACC_IL and AII_PL for binary and multi-class classification.

**Table:** 

**ACC_IL for binary**	**IRV2_Aug**	**IRV2_Raw**	**INV3_Raw**	**CSDCNN_Raw(29)**	**AlexNet_Raw(25)**
IRV2_Aug		1.25	2.75	2.0	4.0
IRV2_Raw			1.5	0.75	2.75
INV3_Raw				−0.75	1.25
CSDCNN_Raw(29)					2.0
AlexNet_Raw(25)	*

**Table:** 

**ACC_PL for binary**	**IRV2 _Aug**	**IRV2 _Raw**	**INV3 _Raw**	**CSDCNN _Raw(29)**	**AlexNet _Raw(25)**	**PFTAS+SVM _Raw(5)**	**PFTAS+QDA _Raw(5)**
IRV2_Aug		2.75	2.0	2.0	4.0	5.0	5.25
IRV2_Raw			−0.75	−0.75	1.25	2.25	2.5
INV3_Raw				0.0	2.0	3.0	3.25
CSDCNN_Raw(29)					2.0	3.0	3.25
AlexNet_Raw(25)						1.0	1.25
PFTAS+SVM_Raw(5)	*						0.25
PFTAS+QDA_Raw(5)	*

**Table:** 

**ACC_IL for multi-class**	**IRV2 _Aug**	**IRV2 _Raw**	**INV3 _Raw**	**CSDCNN _Aug(29)**	**CSDCNN_Raw(29)**	**AlexNet_Aug(29)**	**AlexNet_Raw(29)**	**LetNet_Aug(29)**	**LeNet_Raw(29)**
IRV2_Aug		3.0	4.0	1.0	2.5	4.5	6.0	7.0	8.0
IRV2_Raw			1.0	−2.0	−0.5	1.5	3.0	4.0	5.0
INV3_Raw				−3.0	−1.5	0.5	2.0	3.0	4.0
CSDCNN_Aug(29)					1.5	3.5	5.0	6.0	7.0
CSDCNN_Raw(29)						2.0	3.5	4.5	5.5
AlexNet_Aug(29)							1.5	2.5	3.5
AlexNet_Raw(29)								1.0	2.0
LeNet_Aug(29)	*								1.0
LeNet_Raw(29)	*

**Table:** 

**ACC_PL for multi-class**	**IRV2_Aug**	**IRV2_Raw**	**INV3_Raw**	**CSDCNN_Aug(29)**	**CSDCNN_Raw(29)**	**AlexNet_Aug(29)**	**AlexNet_Raw(29)**	**LetNet_Aug(29)**	**LeNet_Raw(29)**
IRV2_Aug		3.75	3.0	1.0	2.5	4.75	6.0	7.0	8.0
IRV2_Raw			−0.75	−2.75	−1.25	1.0	2.25	3.25	4.25
INV3_Raw				−2.0	−0.5	1.75	3.0	4.0	5.0
CSDCNN_Aug(29)					1.5	3.75	5.0	6.0	7.0
CSDCNN_Raw(29)						2.25	3.5	4.5	5.5
AlexNet_Aug(29)							1.25	2.25	3.25
AlexNet_Raw(29)								1.0	2.0
LeNet_Aug(29)	*								1.0
LeNet_Raw(29)	*								

*† The upper triangle shows the difference between algorithms. The lower triangle shows pairs with statistical significance. Asterisks indicate significant difference between the pairs of algorithms in the table*.

The multiple comparison tests in Table 7 reveal that our breast cancer diagnosis model which uses Inception_ResNet_V2 on the augmented dataset is very powerful. It offers a statistically significant improvement compared to the results from available references that we can find.

This subsection will further compare the experimental results of Inception_ResNet_V2 on histopathological images of breast cancer to those of SVM and 1-NN classifiers with the 1,536-dimension features extracted by the Inception_ResNet_V2 network. Also, it will compare the experimental results of the SVM and 1-NN classifiers with features extracted by other networks.

The experimental results of binary classification of histopathological images of breast cancer with features extracted by Inception_ResNet_V2 are shown in Table S1 in terms of Se, Sp, PPV, DOR, ACC_IL, ACC_PL, F1, AUC and Kappa. Table S2 shows the experimental results of multi-class classification of histopathological images of breast cancer with features extracted by Inception_ResNet_V2 in terms of ACC_IL, ACC_PL, Macro-F1, Micro-F1, and Kappa. Table 8 compared the studies in (5) and ours in terms of ACC_PL, the only evaluation criterion used in (5), when the experimental results are all from SVM and 1-NN classifiers. The differences between our methods and those in (5) are the features. We adopted the Inception_ResNet_V2 network to extract features of histopathological images of breast cancer while those in (5) used other networks to extract features.

**Table 8:** Comparison between different networks extracting features for binary classification/%.

**Criteria**	**Methods**	**Magnification factors**			
		**40X**	**100X**	**200X**	**400X**
ACC_PL	CLBP+SVM_Raw(5)	77.4± 3.8	76.4± 4.5	70.2± 3.6	72.8± 4.9
	GLCM+SVM_Raw(5)	74.0± 1.3	78.6± 2.6	81.9± 4.9	81.1± 3.2
	LBP+SVM_Raw(5)	74.2± 5.0	73.2± 3.5	71.3± 4.0	73.1± 5.7
	LPQ+SVM_Raw(5)	73.7± 5.5	72.8± 5.0	73.0± 6.6	73.7± 5.7
	ORB+SVM_Raw(5)	71.9± 2.3	69.4± 0.4	68.7± 0.8	67.3± 3.1
	PFTAS+SVM_Raw(5)	81.6± 3.0	79.9± 5.4	85.1± 3.1	82.3± 3.8
	IRV2+SVM_Raw	97.93	96.58	97.07	96.62
	**IRV2+SVM_Aug**	**99.27**	**98.97**	**98.90**	**98.74**
	CLBP+1-NN_Raw(5)	73.6± 2.5	71.0± 2.8	69.4± 1.5	70.1± 1.3
	GLCM+1-NN_Raw(5)	74.7± 1.0	76.8± 2.1	83.4± 3.3	81.7± 3.3
	LBP+1-NN_Raw(5)	75.6± 2.4	73.0± 2.4	72.9± 2.3	71.2± 3.6
	LPQ+1-NN_Raw(5)	72.8± 4.9	71.1± 6.4	74.3± 6.3	71.4± 5.2
	ORB+1-NN_Raw(5)	71.6± 2.0	69.3± 2.0	69.6± 3.0	66.1± 3.5
	PFTAS+1-NN_Raw(5)	80.9± 2.0	80.7± 2.4	81.5± 2.7	79.4± 3.9
	IRV2+1-NN_Raw	97.32	95.91	96.12	95.88
	**IRV2+1-NN_Aug**	**98.04**	**97.50**	**97.85**	**97.48**

*† Bold fonts represent the best results among compared approaches with the same classifier*.

The results in the tables in the Supplementary Material show that each classifier gets its best experimental results on the extended datasets of histopathological images of breast cancer, regardless of using binary or multi-class classification. The experimental results of the Inception_ResNet_V2 network on the expanded datasets of histopathological images of breast cancer are the best ones among the results from all of the listed classifiers in the tables in the Supplementary Material. The experimental results of the SVM and 1-NN classifiers are not better than that of the Softmax classifier, even though the features are extracted by the Inception_ResNet_V2 network. Therefore, it is very appropriate to use the Inception_ResNet_V2 network to classify histopathological images of breast cancer.

The results in Table 8 reveal that even when using the same classifiers, such as SVM or 1-NN, the experimental results are different. The results based on the extracted features from the Inception_ResNet_V2 network are much better than those in (5) based on the features extracted by other networks. The best results were also obtained using the extended datasets. This analysis further demonstrates that the deep learning network Inception_ResNet_V2 has a powerful ability to extract informative features automatically.

### Clustering Results

This subsection will describe the great advantages of Inception_ResNet_V2 network when it is used for automatically extracting informative features from histopathological images of breast cancer. The 1,536-dimension features are extracted by using Inception_ResNet_V2 to process histopathological images of breast cancer, and the K-means clustering algorithm is adopted to group these images into proper clusters. In addition, a new AE (Autoencoder) network with a shape of [1536, 500, 2] is constructed to perform a non-linear transformation to the 1,536-dimension feature vectors produced by Inception_ResNet_V2. In this way, the 2-dimension features of the histopathological images of breast cancer can be obtained for K-means in low dimensional space. Here, IRV2+Kmeans represents the clustering results of K-means with the features extracted by Inception_ResNet_V2, while IRV2+AE+Kmeans represents the clustering results of K-means based on the features transformed by our proposed AE using the features extracted by Inception_ResNet_V2.

#### Experiments to Find the Number of Clusters in the Dataset

To find the proper K for K-means, we adopt the internal criterion SSE (Silhouette Score) to search for it. The SSE index combines the degree of condensation and separation and can be used in cases without any label information. The interval of SSE is [−1, 1]. Higher SSE values are associated with samples belonging to the same cluster being closer together and samples belonging to different groups being farther apart. SSE values closer to 1 indicate better clustering.

**Figure 6:**
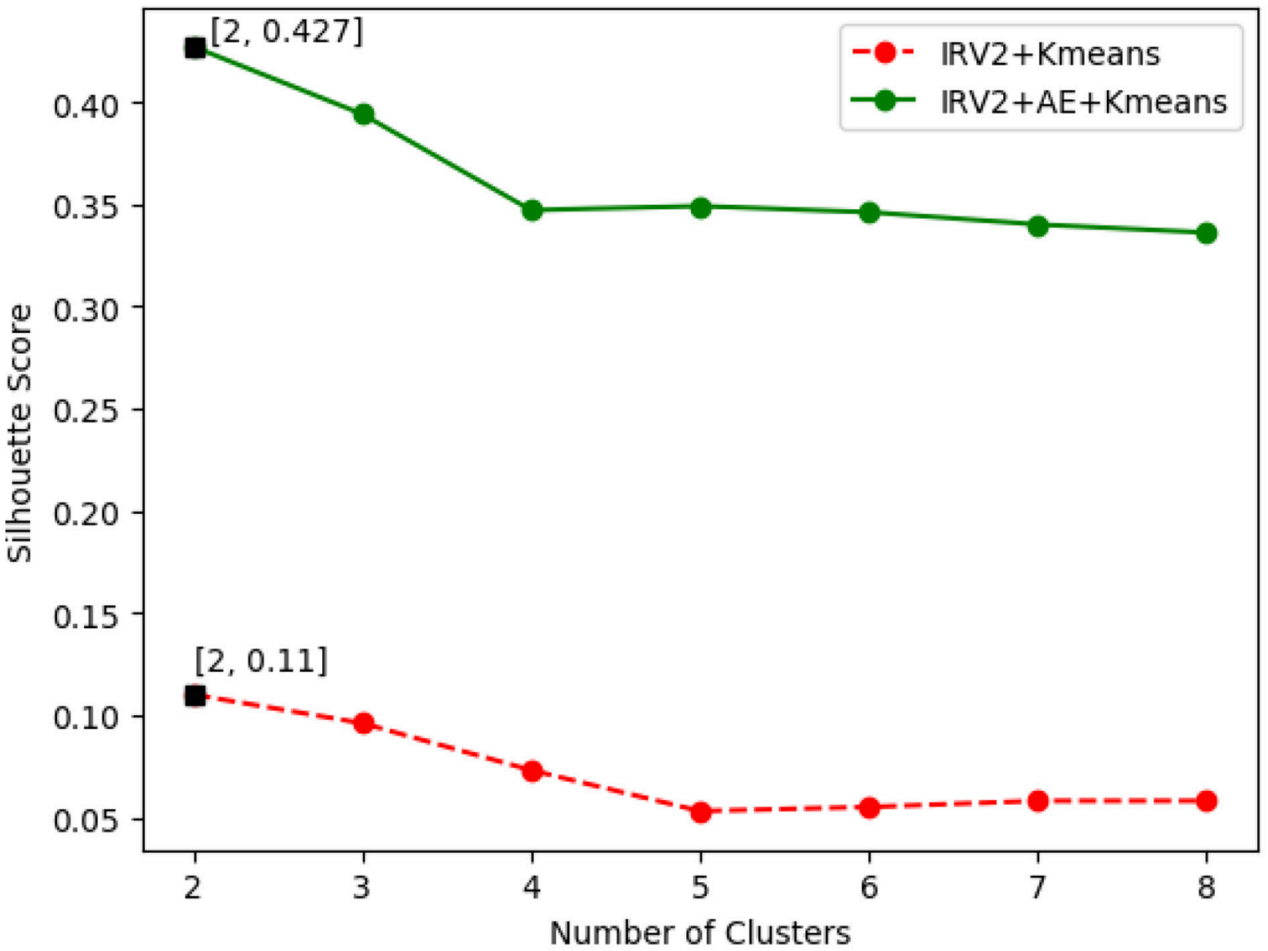
The silhouette score value with different numbers of clusters.

The SSE value of clustering of the histopathological images of breast cancer is variable with the number of clusters. Figure 6 plots the curves of SSE with the number of clusters on the 40X original dataset of histopathological images of breast cancer. The SSE curves of other magnification factor datasets are similar to those in Figure 6.

The results in Figure 6 show the best SSE score was achieved when the number of clusters is 2, regardless of how the features were extracted. This suggests that the histopathological images of breast cancer should be grouped into 2 categories of benign and malignant tumors, which is consistent with the real case. The results in Figure 6 also reveal that the clustering results of IRV2+AE+Kmeans are better than those from IRV2+Kmeans. This means that the proposed AE network can transform the features extracted by the Inception_ResNet_V2 network into much more informative ones, such that a better clustering of histopathological images of breast cancer can be detected.

#### Result Evaluation

**Figure 7:**
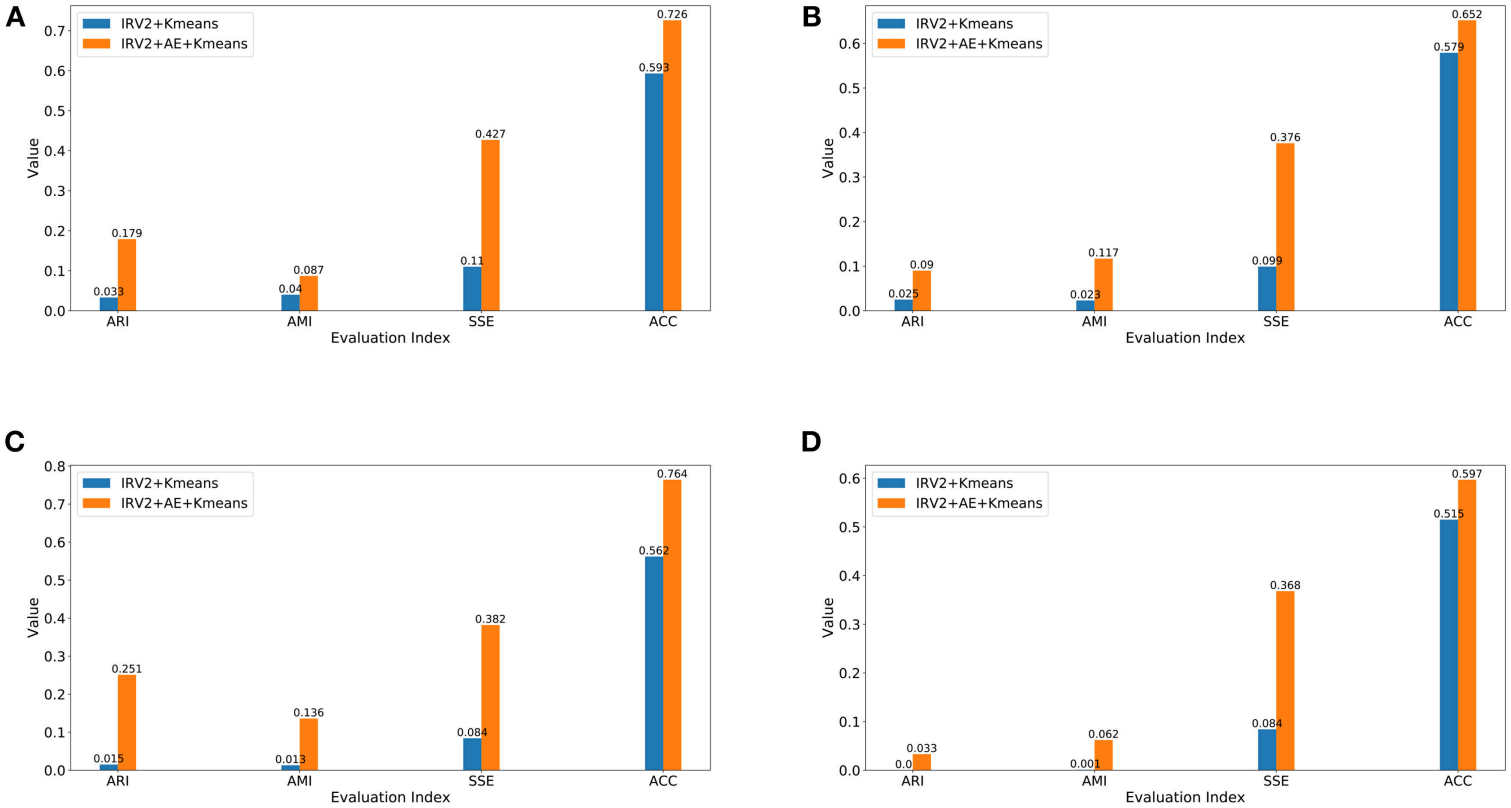
Clustering results in terms of ARI, AMI, SSE, and ACC for datasets with different magnification factors **(A)** 40X, **(B)** 100X, **(C)** 200X, **(D)** 400X.

This subsection will compare the clustering results of IRV2+AE+Kmeans and IRV2+Kmeans in terms of external criteria, including ACC, ARI, AMI, and the internal metric SSE. Figure 7 displays the clustering results in terms of the aforementioned four evaluation criteria on datasets with different magnification factors.

The experimental results in Figure 7 reveal the following facts: (1) the clustering results of IRV2+AE+Kmeans are better than those of IRV2+Kmeans in terms of ARI, AMI, SSE, and ACC on each dataset with different magnification factors. This means that our proposed AE network can produce much more abstract and expressive features by encoding the features extracted by the Inception_ResNet_V2 network. (2) The values of ARI, AMI, SSE, and ACC for the same clustering are ascending, regardless of whether or not any transformation has been applied to the features that were extracted by Inception_ResNet_V2. (3) The best clustering accuracy (ACC) with features produced by the Inception_ResNet_V2 network is 59.3% on the 40X dataset, whereas the best ACC with features transformed by the proposed AE network using extracted features from the Inception_ResNet_V2 network is 76.4% on the 200X dataset. In summary, the best ACC scores of IRV2+AE+Kmeans and IRV2+Kmeans are 76.4 and 59.3%, respectively.

## Conclusions and Future Work

This paper proposed our methods for the analysis of histopathological images of breast cancer based on the deep convolutional neural networks of Inception_V3 and Inception_ResNet_V2 trained with transfer learning techniques. The aforementioned two networks are pre-trained on the large image dataset of ImageNet. Then, their learned structure and parameters are frozen. The number of neurons in the last fully-connected layer is set according to our specific task, and the parameters of the fully-connected layer are re-trained. In this way, the model can be used to perform binary or multi-class classification of the histopathological images of breast cancer. We demonstrate that our experimental results are superior to the ones available in other studies that we have found, and that the Inception_ResNet_V2 network is more suitable for performing analysis of the histopathological images of breast cancer than the Inception_V3 network.

Also, our experimental results from the augmented datasets are much better than those from the original datasets. This is especially true when doing multi-class classification with the histopathological images of breast cancer that we used. Our comparison of the experimental results demonstrates that the Inception_ResNet_V2 network is able to extract much more informative features than the other networks we referenced.

The clustering analysis of the histopathological images of breast cancer using the typical clustering algorithm K-means demonstrates that the proper K value for K-means can be found by using the internal criterion SSE. The proposed AE network can detect much more informative, low dimensional features present in histopathological images of breast cancer. Furthermore, the clustering results produced by K-means using features extracted by Inception_ResNet_V2 and transformed by the proposed AE are much better, in terms of ARI, AMI, SSE, and ACC, than the results produced with features extracted only by Inception_ResNet_V2.

All of the work in this paper demonstrates that the deep convolutional neural network Inception_ResNet_V2 has the advantage when it comes to extracting expressive features from histopathological images of breast cancer. The clustering accuracies of histopathological images of breast cancers are not as good as classification accuracies because the latter used label information.

Finding ways that we can improve the clustering accuracy will require further study. In addition to this, finding the number of clusters of histopathological images of breast cancer in both cases of 8 classes and 2 classes is another task that needs to be addressed.

Noise is a prevalent issue in medical imaging and can have a significant effect on results. Some common sources of noise include white patches on slides after deparaffinization, visible patches on tissue after hydrating, and uneven staining. It was reported that batch effects can lead to huge dissimilarities in features extracted from images (Mathews et al., 2016). For the histopathological images used in this paper, it is a fact that the differences of the resolution, contrast and appearance between images from same class are much more apparent than those from different classes. The variance of the fine-grained histopathological images of breast cancer results in difficulties when trying to classify an image as benign, malignant, or another specific category. How we can avoid or reduce the influence on the analysis of histopathological images of breast cancer from these issues will be the focus of our future work.

## Author Contributions

JX made substantial contributions to the conception and design of the work, drafted the work, and revised it critically for important intellectual content by discussing with CZ, JL, and RL. RL also made substantial contributions to the conception and design of the work. She wrote the code for the algorithms, analyzed the experimental results, and wrote the experimental report. CZ and JL discussed with JX and RL about the technique details, then CZ and JL revised the paper critically for important intellectual content. JX and CZ gave approval for publication of the content. JX and CZ agree to be accountable for all aspects of the work and will ensure that questions related to the accuracy or integrity of any part of the work are appropriately investigated and resolved.

### Conflict of Interest Statement

The authors declare that the research was conducted in the absence of any commercial or financial relationships that could be construed as a potential conflict of interest.
