# Supplementary material for: Deep Learning Based Analysis of Histopathological Images of Breast Cancer
Source: Front Genet. 2019 Feb 19;10:80. doi: 10.3389/fgene.2019.00080 (PMC6390493; doi:10.3389/fgene.2019.00080)
Supplement: Supplementary file 1 [file Data_Sheet_1.DOCX]

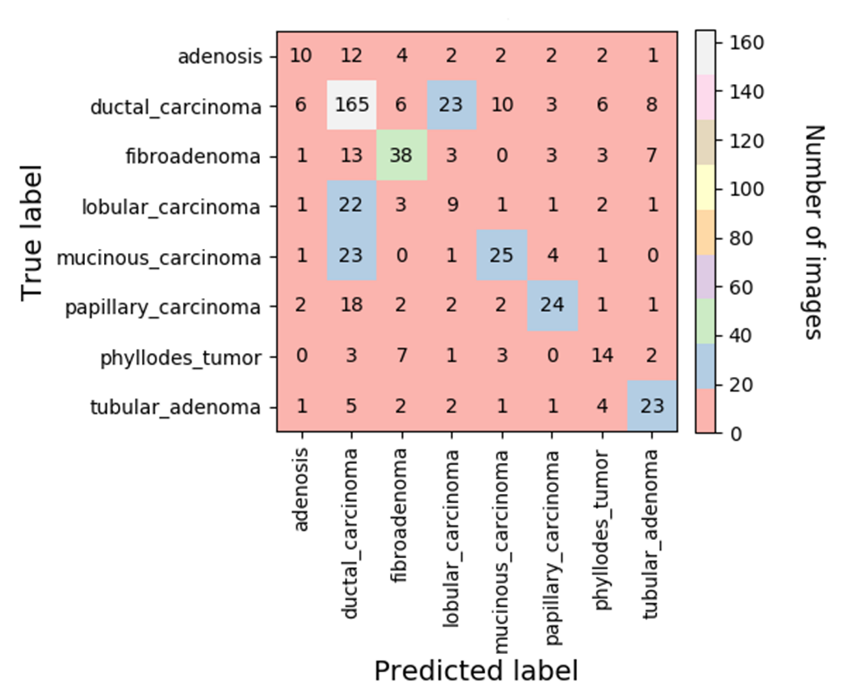


Fig. I The confusion matrix on the dataset with a magnification factor of 400

Table I Results produced by Inception_ResNet_V2 (IRV2) extracting features for binary classification of histopathological images of breast cancer /%

| Network | Dataset | Criteria | Magnification Factors | | | |
| --- | --- | --- | --- | --- | --- | --- |
|  |  |  | 40X | 100X | 200X | 400X |
| IRV2+SVM | Raw_data | Se  Sp  PPV  DOR  ACC_IL | 93.99  88.52  94.90  12065  92.32 | 92.94  84.50  92.72  7178  90.24 | 95.00  79.89  91.51  7549  90.40 | 94.41  74.12  88.97  4841  88.09 |
|  |  | ACC_PL | 97.93 | 96.58 | 97.07 | 96.62 |
|  |  | F1 | 94.44 | 92.83 | 93.22 | 91.61 |
|  |  | AUC | 97.05 | 95.31 | 96.11 | 94.32 |
|  |  | Kappa | 82.01 | 77.54 | 76.77 | 71.17 |
|  | Aug_data | Se  Sp  PPV  DOR  ACC_IL | **97.16**  **98.63**  **98.83**  **245624**  **97.83** | **97.98**  **97.73**  **98.15**  **209530**  **97.87** | **98.01**  **97.24**  **97.66**  **173781**  **97.66** | **97.55**  **97.49**  **97.65**  **154830**  **97.52** |
|  |  | ACC_PL | **99.27** | **98.97** | **98.90** | **98.74** |
|  |  | F1 | **97.98** | **98.07** | **97.83** | **97.60** |
|  |  | AUC | **99.76** | **99.60** | **99.48** | **99.62** |
|  |  | Kappa | **95.63** | **95.70** | **95.28** | **95.04** |
| IRV2+1-NN | Raw_data | Se  Sp  PPV  DOR  ACC_IL | 92.55  85.79  93.67  7499  90.48 | 90.82  80.50  90.82  4086  87.52 | 90.71  78.80  90.71  3632  87.09 | 92.49  69.41  86.02  2793  85.71 |
|  |  | ACC_PL | 97.32 | 95.91 | 96.12 | 95.88 |
|  |  | F1 | 93.11 | 90.82 | 90.71 | 89.97 |
|  |  | AUC | 89.17 | 85.66 | 84.76 | 81.25 |
|  |  | Kappa | 77.75 | 71.32 | 69.52 | 65.23 |
|  | Aug_data | Se  Sp  PPV  DOR  ACC_IL | **96.09**  **99.79**  **99.82**  **1161920**  **97.78** | **95.55**  **98.56**  **98.78**  **146621**  **96.90** | **95.75**  **98.94**  **99.06**  **210223**  **97.22** | **94.69**  **98.69**  **98.72**  **134590**  **96.63** |
|  |  | ACC_PL | **98.04** | **97.50** | **97.85** | **97.48** |
|  |  | F1 | **97.92** | **97.14** | **97.38** | **96.67** |
|  |  | AUC | **97.94** | **97.05** | **97.35** | **96.69** |
|  |  | Kappa | **95.54** | **93.76** | **94.42** | **93.25** |
| **IRV2+Softmax** | Raw_data | Se  Sp  PPV  DOR  ACC_IL | 98.48  96.63  98.46  185774  97.90 | 98.90  92.95  96.45  118782  96.88 | 99.13  92.80  96.39  147138  96.98 | 98.06  92.10  96.51  58835  96.98 |
|  |  | ACC_PL | 98.03 | 97.07 | 82.74 | 88.12 |
|  |  | F1 | 98.47 | 97.66 | 97.74 | 97.28 |
|  |  | AUC | 99.57 | 98.84 | 99.61 | 98.81 |
|  |  | Kappa | 95.12 | 92.96 | 93.18 | 91.05 |
|  | **Aug_data** | Se  Sp  PPV  DOR  ACC_IL | **99.95**  **99.61**  **99.66**  **56122884**  **99.79** | **99.45**  **99.26**  **99.39**  **2440736**  **99.37** | **99.65**  **99.18**  **99.31**  **3427114**  **99.43** | **98.88**  **99.34**  **99.42**  **1342245**  **99.10** |
|  |  | ACC_PL | **99.93** | **99.96** | **100.0** | **99.90** |
|  |  | F1 | **99.81** | **99.42** | **99.48** | **99.15** |
|  |  | AUC | **100.0** | **99.99** | **99.95** | **99.97** |
|  |  | Kappa | **99.59** | **98.72** | **98.86** | **98.19** |

^🕆^ Bold font represents better scores for a given metric obtained by each classifier, and a red underline represents the best score for each metric out of all the classifiers (Table II uses the same format).

Table II Results produced by Inception_ResNet_V2 (IRV2) extracting features for multi-class classification of histopathological images of breast cancer /%

| Network | Dataset | Criteria | Magnification Factors | | | |
| --- | --- | --- | --- | --- | --- | --- |
|  |  |  | 40X | 100X | 200X | 400X |
| IRV2+SVM | Raw_data | ACC_IL | 77.30 | 75.36 | 70.53 | 71.61 |
|  |  | ACC_PL  Macro-F1  Micro-F1  Kappa | 92.56  73.68  77.30  70.33 | 92.03  69.27  75.36  66.60 | 90.50  64.99  70.53  60.80 | 91.22  66.13  71.61  61.80 |
|  | Aug_data | ACC_IL | **95.42** | **94.45** | **94.24** | **94.73** |
|  |  | ACC_PL  Macro-F1  Micro-F1  Kappa | **95.68**  **95.38**  **95.42**  **94.75** | **95.20**  **94.45**  **94.45**  **93.63** | **95.52**  **94.21**  **94.24**  **93.41** | **95.73**  **94.57**  **94.73**  **93.97** |
| IRV2+1-NN | Raw_data | ACC_IL | 68.78 | 66.88 | 63.25 | 56.41 |
|  |  | ACC_PL  Macro-F1  Micro-F1  Kappa | 88.82  64.56  68.78  60.04 | 89.98  59.66  66.88  55.84 | 88.59  57.00  63.25  51.74 | 86.94  48.40  56.41  42.27 |
|  | Aug_data | ACC_IL | **94.79** | **92.64** | **92.63** | **92.15** |
|  |  | ACC_PL  Macro-F1  Micro-F1  Kappa | **93.78**  **94.57**  **94.79**  **94.03** | **92.41**  **92.20**  **92.64**  **91.57** | **93.88**  **92.45**  **92.63**  **91.56** | **93.16**  **91.60**  **92.15**  **91.01** |
| **IRV2+Softmax** | Raw_data | ACC_IL | 92.07 | 88.06 | 87.62 | 84.50 |
|  |  | ACC_PL  Macro-F1  Micro-F1  Kappa | 89.11  90.89  92.07  89.74 | 88.45  85.67  88.06  84.03 | 86.07  84.08  87.62  82.84 | 71.42  80.13  84.50  79.70 |
|  | **Aug_data** | ACC_IL | **97.63** | **97.00** | **96.89** | **97.49** |
|  |  | ACC_PL  Macro-F1  Micro-F1  Kappa | **98.42**  **97.68**  **97.63**  **97.28** | **98.07**  **97.06**  **97.00**  **96.55** | **97.85**  **97.02**  **96.89**  **96.44** | **97.40**  **97.48**  **97.49**  **97.13** |
